# Oats in the Diet of Children with Celiac Disease: Preliminary Results of a Double-Blind, Randomized, Placebo-Controlled Multicenter Italian Study

**DOI:** 10.3390/nu5114653

**Published:** 2013-11-20

**Authors:** Simona Gatti, Nicole Caporelli, Tiziana Galeazzi, Ruggiero Francavilla, Maria Barbato, Paola Roggero, Basilio Malamisura, Giuseppe Iacono, Andrea Budelli, Rosaria Gesuita, Carlo Catassi, Elena Lionetti

**Affiliations:** 1 Department of Pediatrics, Università Politecnica delle Marche, 60121 Ancona, Italy; E-Mails: nicole.capo@hotmail.it (N.C.); t.galeazzi@univpm.it (T.G.); catassi@tin.it (C.C.); 2 Interdisciplinary Department of Medicine, University of Bari, 70124 Bari, Italy; E-Mail: rfrancavilla@gmail.com; 3 Department of Pediatrics, “Sapienza” University of Rome, 00161 Roma, Italy; E-Mail: Maria.Barbato@uniroma1.it; 4 Neonatal Intensive Care Unit, Department of Clinical Sciences and Community Health, Fondazione IRCCS Ca’ Granda Ospedale Maggiore Policlinico, University of Milan, 20122 Milano, Italy; E-Mail: paola.roggero@unimi.it; 5 Department of Pediatrics, S. Maria dell’Olmo Hospital Cava de’ Tirreni, 84013 Salerno, Italy; E-Mail: basiliomalamisura@libero.it; 6 Pediatric Gastroenterology Unit, “G. Di Cristina” Children Hospital, 90134 Palermo, Italy; E-Mail: stoai@inwind.it; 7 R&D Heinz Italia S.p.A, 04100 Latina, Italy; E-Mail: Andrea.Budelli@it.hjheinz.com; 8 Department of Epidemiology, Biostatistics and Medical Information Technology, Università Politecnica delle Marche, 60121 Ancona, Italy; E-Mail: r.gesuita@univpm.it; 9 Department of Pediatrics, Università di Catania, 95123, Catania, Italy; E-Mail: elenalionetti@inwind.it

**Keywords:** : oats, celiac disease, gluten-free diet, intestinal permeability, gastrointestinal symptoms

## Abstract

A gluten-free diet (GFD) is currently the only available treatment for patients with celiac disease (CD). Several clinical trials have demonstrated that most celiac patients can tolerate a medium-high quantity of oats without any negative clinical effects; however, the inclusion of oats in GFD is still a matter of debate. In this study, Italian children with CD were enrolled in a 15-month, randomized, double-blind, placebo-controlled multicenter trial. Participants were randomized in two groups following either A-B treatment (6 months of diet “A”, 3 months of standard GFD, 6 months of diet “B”), or B-A treatment (6 months of diet “B”, 3 months of standard GFD, 6 months of diet “A”). A and B diets included gluten-free (GF) products (flour, pasta, biscuits, cakes and crisp toasts) with either purified oats or placebo. Clinical data (Gastrointestinal Symptoms Rate Scale [GSRS] score) and intestinal permeability tests (IPT), were measured through the study period. Although the study is still blinded, no significant differences were found in GSRS score or the urinary lactulose/mannitol (L/M) ratio between the two groups after 6 months of treatment. These preliminary results suggest that the addition of non-contaminated oats from selected varieties in the treatment of children with CD does not determine changes in intestinal permeability and gastrointestinal symptoms.

## 1. Introduction

Celiac disease (CD) is an immune-mediated disorder, triggered in genetically susceptible individuals by ingested gluten, the alcohol-soluble complex present in wheat, rye, and barley. The clinical spectrum of CD is extremely variable, including (a) typical CD, with the classical features of intestinal malabsorption; (b) atypical CD (characterized by extra-intestinal manifestations); (c) silent CD, (occasionally found following serological screening in subjects who are asymptomatic); (d) potential CD, showing positivity of celiac serology associated with a normal (or nearly normal) intestinal mucosa at the small intestinal biopsy. The cornerstone of treatment is the lifelong exclusion of gluten-containing cereals from the diet, the gluten-free diet (GFD). 

Oats were originally excluded from the diet of people with CD. However, evidence supporting the toxicity of oats for CD individuals was poor. More recently, several studies have shown that medium-high amounts of gluten-uncontaminated oats can be safely ingested by patients with CD. In 1995, Finnish investigators compared the effect of 50–70 g/day of oats to placebo in 92 adults with CD on a GFD at diagnosis or in follow-up. They found no difference in clinical and laboratory outcomes and, more importantly, there was no sign of histological damage after 12 months of an oat-containing GFD [1]. These data have been replicated in other studies conducted in adults and children affected with CD or dermatitis herpetiformis [2,3,4,5,6,7,8,9,10,11], thereby confirming the safety of oat-based products for CD patients, provided that gluten contamination is avoided in the production chain. Recent studies suggest that some oats varieties may show a degree of residual toxicity *in vitro*, suggesting that there are differences between oat varieties in relation to their safety/toxicity for people with CD [12,13,14,15,16].

Official recommendations acknowledge the safety of products containing purified oats, and several national associations for CD allow inclusion of oats in the diet of people with CD [17,18]. The European Commission Regulation No. 41/2009 has included oats among allowed ingredients when the gluten content does not exceed 20 parts per million (ppm). During the last years, thousands of CD patients have been consuming large amounts of oat-based products in Northern European countries and Canada without any reported major side effects.

Oat is not a staple food in the diet of Mediterranean populations. This is probably the main reason why an oats “resurrection” in the GFD has not raised immediate interest in Southern European countries. In Italy, products containing gluten-uncontaminated oats are not currently available. This situation will hopefully change in the near future; rich in soluble dietary fiber, vitamins and minerals, the inclusion of oats unquestionably improves the nutritional value and increases the palatability of the GFD, while expanding food choices and ultimately improving the quality of life for people with CD [17]. 

For these reasons, we undertook a prospective, multicenter investigation on the safety and acceptance of gluten-free (GF) oat-based products from selected oat varieties in the diet of Italian CD children in treatment with the GFD. Clinical monitoring during the study was based on: (a) score of intestinal symptoms, (b) serological CD markers (IgA class anti-transglutaminase antibody), and (c) results of the double sugar intestinal permeability test (IPT), as a marker of mucosal integrity of the small intestine. In this work, we present the preliminary clinical and intestinal permeability results of this multicenter, placebo-controlled and double-blind study. 

## 2. Patients and Methods

### 2.1. Study Population

Children (age range: 4–14 years) with biopsy-proven diagnosis of CD, on a GFD for at least 2 years, were recruited in 7 different Pediatric Gastroenterology Services in Italy (Ancona, Bari, Catania, Monza, Palermo, Roma, Cava de’ Tirreni). Patients who (1) have other chronic conditions (including type 1 diabetes or inflammatory bowel disease), or (2) did not adhere to the GFD (as demonstrated by elevation of serological markers at enrollment) or (3) were on a GFD for less than 2 years were excluded. 

### 2.2. Study Design and Diets

**Figure 1:**
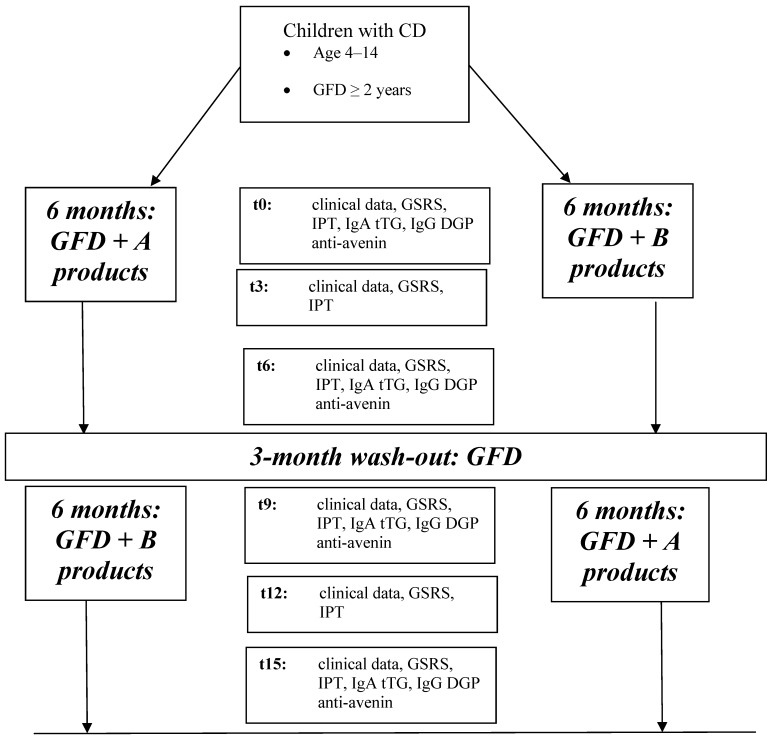
Flow-chart of the multicenter trial (t0: baseline, t3: 3-month follow-up, t6: 6-month follow-up, t9: 9-month follow-up, t12: 12-month follow-up, t15: 15-month follow-up; GSRS: Gastrointestinal Symptoms Rate Scale, IgA tTG: IgA class anti-transglutaminase antibody, IgG DGP: IgG class deamidated gliadin peptides antibody).

The protocol of the study is shown in Figure 1. 

Clinical data (gastrointestinal [GI] symptoms, growth data) and IPT by measurement of urinary lactulose/mannitol (L/M) ratio were monitored at 0, 3, 6, 9, 12 and 15 months. Serological (IgA-antitransglutaminase-TTG, IgG-deamidated gliadin peptide-DGP, and anti-avenin antibodies) and biochemical data were measured at 0, 6, 9, and 15 months. An accurate food diary was completed in the 3 days preceding each visit at 3, 6, 12 and 15 months. Symptoms and/or side effects related to the ingestion of the products under investigation were promptly recorded and the decision to continue or withdraw from the study protocol was made after discussion with the children and their families. 

In this preliminary report, we describe the GI symptoms and IPT findings after 6 months of participation in this study.

### 2.3. Methods

The occurrence of GI symptoms was monitored through the Gastrointestinal Symptoms Rate Scale (GSRS) at 0, 3, 6, 9, 12 and 15 months.

The IPT was performed as previously described [19]. After an overnight fast and bladder emptying, an oral solution containing 5 g of lactulose and 2 g of mannitol was administered. Urine was collected during the following 5 h. An aliquot was preserved at −20 °C with sodium azide. Urinary excretion of each sugar was assessed using a high performance anion-exchange Chromatography (Dionex DX-500). The ratio of recovered to ingested sugar was reported as ratio of lactulose% to mannitol% (L/M). According to our own reference values, a urinary L/M ratio > 0.08 was considered abnormal (data not published). All IPTs were performed in the Laboratory of the Department of Pediatrics, Università Politecnica delle Marche, Ancona.

### 2.4. Ethics

Patients and families received appropriate information and informed consent was obtained. The protocol was approved by the Ethical Committee of Università Politecnica delle Marche, Ancona. The trial was registered on www.clinicaltrials.gov (identifier: NCT00808301). 

### 2.5. Statistical Analysis

Statistical analyses were performed using GraphPad Prism version 5 (GraphPad Software, San Diego, CA, USA). Non-parametric tests were used because variables were not normally distributed and did not have equal variance. Results are presented as median (interquartile range [IQR]). Differences between groups were assessed by the Mann-Whitney U test. The Wilcoxon matched pairs test was used to calculate the differences for pair data. A *p* value < 0.05 was considered significant.

## 3. Results

Overall, 306 children (group A-B: 154, group B-A: 152) were enrolled in the study (median age = 9.62, IQR = 7.23–11.9 years). Patients’ enrollment was concluded in March 2013. Fifty-five out of 154 patients enrolled in group A-B (35.7%) and 42/152 patients in group B-A (27.6%) dropped out from the study within the first 6 months of treatment (*p* = 0.14). One hundred-seventy one children received at least 6 months of treatment, 75 patients received A treatment (group A), and 96 received B treatment (group B). Clinical and biochemical data from these subgroups were considered for the purpose of this preliminary analysis. 

Table 1 summarizes clinical and demographic characteristics of the study-group at enrollment.

**Table 1:** Clinical and demographic features of children in group A and B at enrollment. M = males, F = females, IQR = interquartile range.

	Group A (*N* = 75)	Group B (*N* = 96)	*p*
Gender distribution (M:F)	1:2.5	1:1.9	0.50
Age at diagnosis Median (IQR)	3.48 (1.98–6.36)	2.84 (1.83–6.03)	0.38
Age at enrollment Median (IQR)	8.76 (7.07–11.38)	9.35 (7.24–12.01)	0.54
Duration of diet Median (IQR)	4.25 (2.11–6.04)	4.49 (1.25–6.55)	0.06
GSRS score at enrollment Median (IQR)	3 (0–5.25)	2 (0–4.5)	0.36
L/M at enrollment Median (IQR)	0.055 (0.030–0.083)	0.052 (0.026–0.088)	0.62

**Figure 2:**
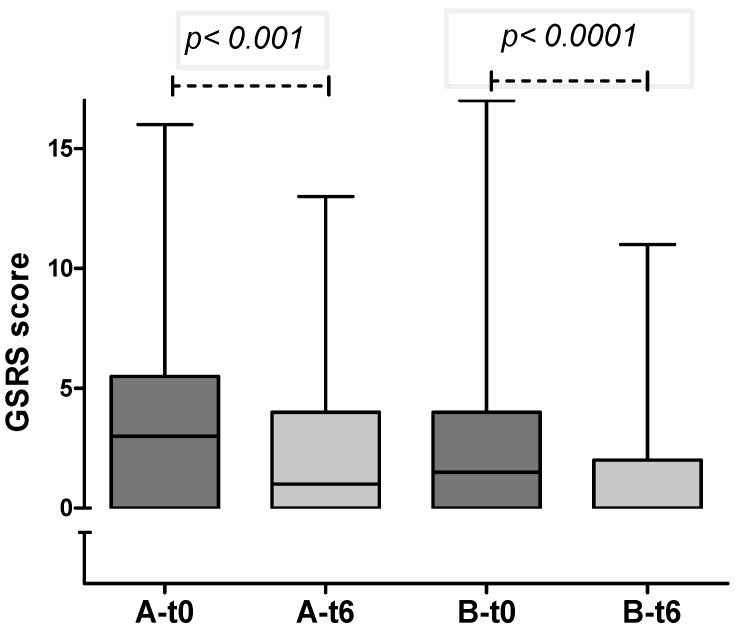
GSRS score in group A and B at enrollment and after 6 months (median and IQR): in both groups a significant reduction in GI symptoms was observed.

**Figure 3:**
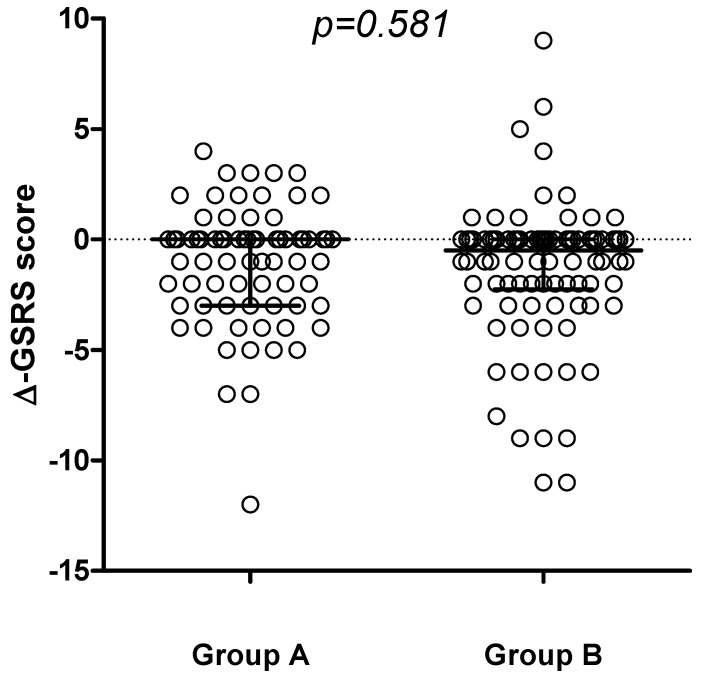
Change in GSRS score (∆-GSRS score, median and IQR) between t6 and t0 in the 2 groups.

In both groups, a significant decrease in gastrointestinal symptoms was recorded through the 6-months period (Figure 2), with no differences detected comparing the delta-GSRS score (Δ-GSRS score) between the 2 groups (Figure 3).

**Figure 4:**
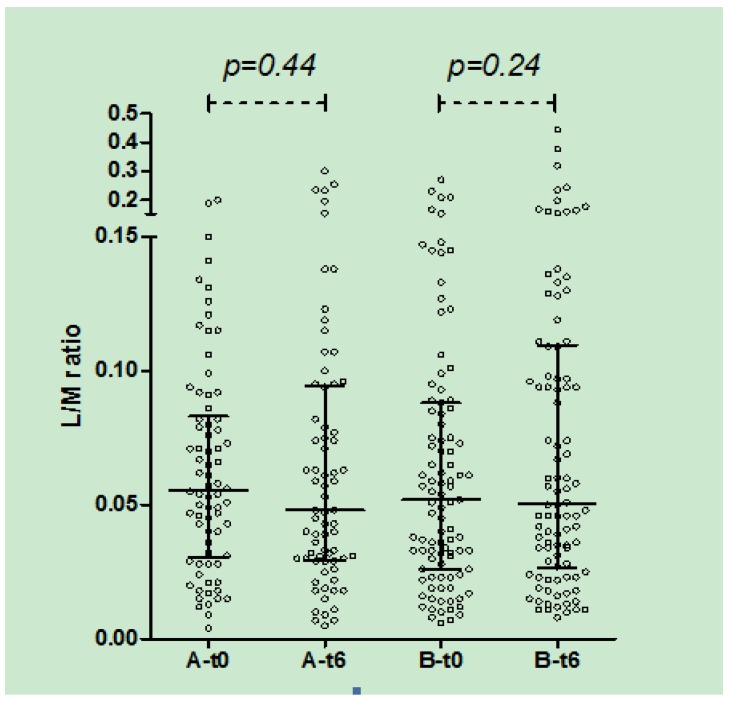
Urinary L/M ratio in group A and B at t0 and t6 (median, IQR): in both groups no significant difference was observed after 6 months of treatment.

In both A and B groups, there was no significant change in urinary L/M ratio after 3 (data not shown) and 6 months of treatment (Figure 4).

**Figure 5:**
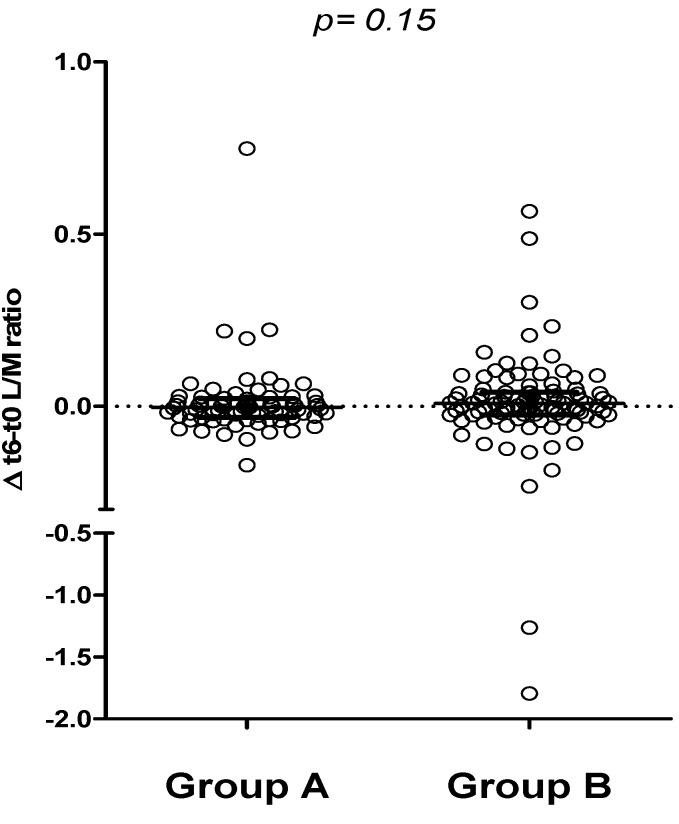
Comparison between t6 and t0 urinary L/M values in groups A and B (∆ t6-t0, median and IQR).

Furthermore, comparing the 6-month change in urinary L/M ratio (Δ-L/M), no difference was found between groups A and B, suggesting no relevant impact of a 6-month period of either oats or placebo-added GFD on the intestinal permeability test (Figure 5).

**Figure 6:**
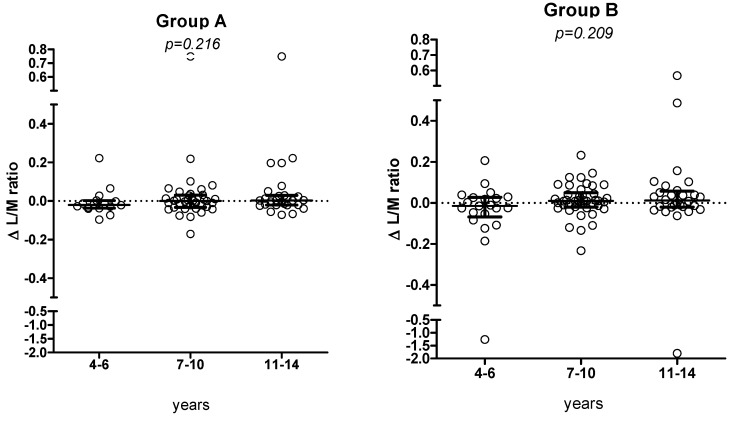
Changes in L/M values (median, IQR) according to the 3 age classes, in groups A and B, respectively.

The 6-month Δ L/M value did not show a significant trend according to the different age groups (corresponding to different amount of oats or placebo) (Figure 6a,b).

## 4. Discussion

In a large group of CD children that had not previously consumed oats, we found that the prolonged intake of a considerable amount of daily oats did not determine any relevant change in terms of clinical symptoms and intestinal permeability, even though the results were analyzed in a blind fashion.

Gastrointestinal symptoms were evaluated using the GSRS, that is an interview-based rating scale consisting of 15 items, validated and widely used for assessment of GI symptoms both in adults and children [20,21,22,23,24]. In both groups, there was a significant reduction in GI symptoms during the 6-month period of observation. Such a finding could simply reflect the effect of “being enrolled in a trial.” The addition of non-contaminated oats from selected varieties in one of the two groups apparently had no impact on this clinical trend. Dyspeptic symptoms (described in other studies as related to the high amount of fiber in oats) were not recorded in our population. Although the number of dropouts was not significantly different in group A and B during the first 6 months of dietary intervention, the final analysis of this study, including reasons for withdrawal, will clarify the possible impact of oats ingestion on GI symptoms.

Besides a few exceptional cases reported in the literature [25], previous studies showed that the prolonged administration of oats does not induce any mucosal change at the intestinal level. Since performing repeated follow-up small intestinal biopsies was deemed unethical in our study, we evaluated the mucosal integrity by a non-invasive procedure, *i.e.*, the IPT. The integrity of the intestinal barrier can be evaluated by the IPT, by quantifying the amount of orally administered substances recovered in urine. Mannitol (monosaccharide) and lactulose (disaccharide) are largely used as permeability probes due to their different intestinal permeability index. Mannitol is absorbed through enterocyte membrane’s pores (transcellular pathway), while lactulose is absorbed through tight junctions (paracellular pathway). Ratio between the percentage of lactulose and mannitol excretion represents the permeability index that reflects the intestinal epithelium integrity. The non-invasive functional IPT is a sensitive tool for both triage of active celiac disease, as well as for monitoring celiac patients on a GFD [26]. In patients with active CD, a sugar “paradox” pathway is usually observed: the amount of urinary recovered mannitol is decreased due to the small intestine reduced absorptive surface (villous atrophy), while urinary recovered lactulose is increased because of damage of the TJs and consequent widening of paracellular spaces. For these reasons, an increased excretion disaccharide/monosaccharide ratio is the usual finding associated with CD damage of the small intestinal mucosa. In adults with CD, the IPT has been shown to be more sensitive than serological methods in monitoring patients during follow-up and detecting minimal changes related to gluten ingestion [27,28]. Conversely, the same test was not a reliable instrument to detect asymptomatic CD subjects in a mass screening project [29]. Furthermore, IPT did not perform optimally in two recent trials of larazotide acetate in measuring changes in intestinal permeability after a 14-day [30] or 6-week [31] gluten challenge.

In our study, IPT was chosen as a non-invasive surrogate measure to detect minimal damages of the small intestinal mucosa at different time points. Thus far, no study has been performed to investigate the effects of prolonged oats ingestion on the intestinal permeability test in patients with CD. We did not find significant changes in urinary L/M ratio either in group A or B. This finding is further supported by the comparison of the Δ L/M in the two groups, again failing to show any difference between groups. However, the final results of our large study will give more information about the possible effect of oats ingestion on the results of the intestinal permeability. More data will also be available on the correlation between permeability indexes and serological CD markers. 

The daily amount of suggested intake of oats products was calculated to guarantee medium-high amounts of oats (up to 40 g/day for older children). Although it was not possible to analyze the data according to the amount of ingested oats, the evaluation of the IPT results in the three age groups (corresponding to the different quantity of study products suggested) again showed no significant differences between groups. Overall, the lack of changes in intestinal permeability suggests that the varieties of gluten-uncontaminated oats chosen for production of these oat-based items are safe. The sample analyzed for the purpose of this analysis represents more than 50% of the enrolled study group. However, this analysis has not considered the original cross-over design of the protocol. Such a limitation implies caution in the interpretation of the results.

## 5. Conclusions

The preliminary results of this investigation suggest that the oats varieties used for producing the gluten-uncontaminated products used in this study are safe when administered for a 6-month period of time. These preliminary conclusions are based on both clinical data and the results of a non-invasive test for intestinal mucosa barrier function (IPT). The final analysis of the data collected in this study will provide conclusive data on the safety and the acceptance of these oat-based products in Italian children in treatment with the GFD.
